# Artificial intelligence-based radiographic extent analysis to predict tuberculosis treatment outcomes: a multicenter cohort study

**DOI:** 10.1038/s41598-024-63885-0

**Published:** 2024-06-07

**Authors:** Hyung-Jun Kim, Nakwon Kwak, Soon Ho Yoon, Nanhee Park, Young Ran Kim, Jae Ho Lee, Ji Yeon Lee, Youngmok Park, Young Ae Kang, Saerom Kim, Jeongha Mok, Joong-Yub Kim, Doosoo Jeon, Jung-Kyu Lee, Jae-Joon Yim

**Affiliations:** 1 Division of Pulmonary and Critical Care Medicine, Department of Internal Medicine, Seoul National University Bundang Hospital, Seongnam, Republic of Korea; 2 Department of Internal Medicine, Seoul National University College of Medicine, Seoul, Republic of Korea; 3 Division of Pulmonary and Critical Care Medicine, Department of Internal Medicine, Seoul National University College of Medicine, Seoul National University Hospital, Seoul, Republic of Korea; 4 Department of Radiology, Seoul National University College of Medicine, Seoul National University Hospital, Seoul, Republic of Korea; 5 Medical Research Collaborating Center, Seoul National University Hospital, Seoul National University College of Medicine, Seoul, Republic of Korea; 6 Division of Clinical Research, International Tuberculosis Research Center, Seoul, Republic of Korea; 7 Division of Pulmonary and Critical Care Medicine, Department of Internal Medicine, National Medical Center, Seoul, Republic of Korea; 8 Division of Pulmonary and Critical Care Medicine, Department of Internal Medicine, Severance Hospital, Yonsei University College of Medicine, Seoul, Republic of Korea; 9 Division of Pulmonology, Allergy and Critical Care Medicine, Department of Internal Medicine, Pusan National University Hospital, Busan, Republic of Korea; 10 Department of Internal Medicine, Pusan National University School of Medicine, Busan, Republic of Korea; 11 Department of Internal Medicine, Pusan National University Yangsan Hospital, Yangsan, Republic of Korea; 12 Department of Internal Medicine, Pusan National University School of Medicine, Yangsan, Republic of Korea; 13 Division of Pulmonary and Critical Care Medicine, Department of Internal Medicine, Seoul Metropolitan Government-Seoul National University Boramae Medical Center, Seoul, Republic of Korea

**Keywords:** Tuberculosis, Pulmonary, Artificial intelligence, Radiography, Thoracic, Treatment outcome, Respiratory tract diseases, Infectious diseases, Tuberculosis

## Abstract

Predicting outcomes in pulmonary tuberculosis is challenging despite effective treatments. This study aimed to identify factors influencing treatment success and culture conversion, focusing on artificial intelligence (AI)-based chest X-ray analysis and Xpert MTB/RIF assay cycle threshold (Ct) values. In this retrospective study across six South Korean referral centers (January 1 to December 31, 2019), we included adults with rifampicin-susceptible pulmonary tuberculosis confirmed by Xpert assay from sputum samples. We analyzed patient characteristics, AI-based tuberculosis extent scores from chest X-rays, and Xpert Ct values. Of 230 patients, 206 (89.6%) achieved treatment success. The median age was 61 years, predominantly male (76.1%). AI-based radiographic tuberculosis extent scores (median 7.5) significantly correlated with treatment success (odds ratio [OR] 0.938, 95% confidence interval [CI] 0.895–0.983) and culture conversion at 8 weeks (liquid medium: OR 0.911, 95% CI 0.853–0.973; solid medium: OR 0.910, 95% CI 0.850–0.973). Sputum smear positivity was 49.6%, with a median Ct of 26.2. However, Ct values did not significantly correlate with major treatment outcomes. AI-based radiographic scoring at diagnosis is a significant predictor of treatment success and culture conversion in pulmonary tuberculosis, underscoring its potential in personalized patient management.

## Introduction

Tuberculosis remains a major global health challenge, with 10 million new cases annually and 1.3 million deaths each year, making it the second leading cause of death from a single infectious agent^1^. The advent of anti-tuberculosis drugs in the 1940s significantly reduced TB mortality rates, and the current four-drug regimen (isoniazid, rifampin, ethambutol, and pyrazinamide) boasts an 85% treatment success rate.

Predicting outcomes in pulmonary tuberculosis, however, is complex. Factors like age, body mass index (BMI), nutritional status, alcohol and tobacco use, diabetes, previous tuberculosis history, and acquired immune deficiency syndrome impact treatment results^2–4^. The diversity of these factors complicates comprehensive clinical assessment.

Radiographic findings play a crucial role in managing drug-susceptible tuberculosis. Imaging variations, especially the presence or absence of cavities, significantly influence treatment duration^5,6^. While chest X-ray findings, including cavities, can be visually quantified and correlate with disease burden reduction during treatment, such assessments are often subjective and vary between observers^7^. Recent advancements in artificial intelligence (AI) have led to the development of AI-based models that calculate a tuberculosis activity score from chest X-rays, potentially serving as an indicator of severity classification^8^.

The Xpert *Mycobacterium tuberculosis* (MTB)/resistance to rifampicin (RIF) assay, a hemi-nested polymerase chain reaction (PCR) assay targeting the *Mycobacterium tuberculosis rpoB* gene^9,10^, is used for rapid TB diagnosis and rifampin resistance detection. Its cycle threshold (Ct) value offers a semi-quantitative measure of bacillary burden in sputum, with a one-cycle increase indicating a 50% reduction in MTB DNA^10–12^.

This study aims to assess the prognostic value of AI-based TB activity scores and Xpert MTB/RIF assay Ct values in predicting treatment outcomes in patients with rifampin-susceptible pulmonary TB.

## Materials and methods

### Eligibility criteria

This retrospective study, spanning January 1 to December 31, 2019, involved six South Korean referral centers. The selection of these centers was strategic, designed to ensure coverage of each major region within the country. Adults (age > 19) diagnosed with rifampicin-susceptible pulmonary tuberculosis, confirmed via positive Xpert MTB/RIF assay results from self-expectorated sputum samples, were included. Exclusions were patients without pre-treatment chest posteroanterior images, those tested post-treatment, or with missing Xpert MTB/RIF assay Ct values.

### Data collection

Patient demographics, including age, sex, BMI, smoking, and drinking habits, plus comorbidities, were reviewed from medical records. The Xpert MTB/RIF assay, manufactured by Cepheid (Sunnyvale, CA, USA), was employed on sputum samples following the manufacturer's guidelines. This involved the use of five rpoB probes within a real-time PCR setup to identify tuberculosis and determine rifampicin resistance^10,12^. Median and minimum cycle threshold (Ct) values from the assay were extracted and utilized as markers to gauge the disease's severity at diagnosis^13,14^.

### Radiographic analysis

Pulmonary tuberculosis extent was quantified using an AI-based prognostic model (DeepCatch X TB v1.0, MEDICAL IP Co., Ltd., Seoul, Korea), derived and validated from 6654 chest X-rays, exhibiting an area under the receiver operating characteristic curve (AUC) of 0.83–0.84 in an external test set^8,15^. The model’s original output was the probability of active tuberculosis (between 0 and 1), and a value higher than the threshold of 0.16 achieved 95% sensitivity for discriminating active tuberculosis from healed disease. Chest X-ray images, downloaded as Digital Imaging and Communications in Medicine files from each center, were analyzed to calculate the tuberculosis extent. The X-ray area with a probability higher than 0.16 was divided by the entire lung parenchyma area. Following the authorization from the company, the model was made available online at https://tisepx.com/. It is important to note that Xpert Ct values were not incorporated into the development of the model.

Cavities were identified by radiologists and investigators through formal reading and inspection. Data were recorded on standardized forms and the study was registered at clinicaltrials.gov (NCT05477966), with approval from institutional review boards / ethics committees of each center (Supplementary Table S1). The requirement for informed consent was waived due to the retrospective study design. This study was conducted according to the principles of the Declaration of Helsinki.

### Outcome definitions

Treatment outcomes were categorized as 'treatment success' or 'unfavorable outcome' per World Health Organization definitions^16,17^. A bacteriologically confirmed patient was considered cured upon completing treatment, as recommended by national policy, showing a bacteriological response without evidence of failure. Treatment failure was defined as a positive sputum culture at month 5 or later of treatment. 'Treatment success' included patients cured or completing treatment without failure; others were 'unfavorable'.

Culture conversion at 8 weeks post-treatment was also analyzed. Culture conversion was defined as two consecutive negative cultures^17^, with the first day of a negative result marked as the day of conversion.

### Statistical analysis

Continuous variables were presented as medians and interquartile ranges (IQRs), analyzed using independent t-tests or Wilcoxon rank-sum tests. Categorical variables were compared using chi-square or Fisher’s exact tests. Logistic regression calculated odds ratios (ORs) and 95% confidence intervals (CIs) for outcome factors, with restricted cubic spline for linearity testing^18–20^. Multivariate analysis included variables significant at a 20% level in univariate analysis, using stepwise selection retaining variables that were significant at the 5% level for multicollinearity. Entry and removal conditions during stepwise selection were set at *P* < 0.05 and *P* > 0.10, respectively. For rare categories, a penalized maximum-likelihood method (Firth correction) was used to estimate the ORs^21,22^, with the CIs calculated using the profile penalized likelihood approach. Significance was assessed using penalized likelihood ratio tests. SAS software (SAS for Windows, version 9.4; SAS institute, Cary, NC, US) was used for all statistical analyses.

## Results

### Patient selection

**Figure 1:**
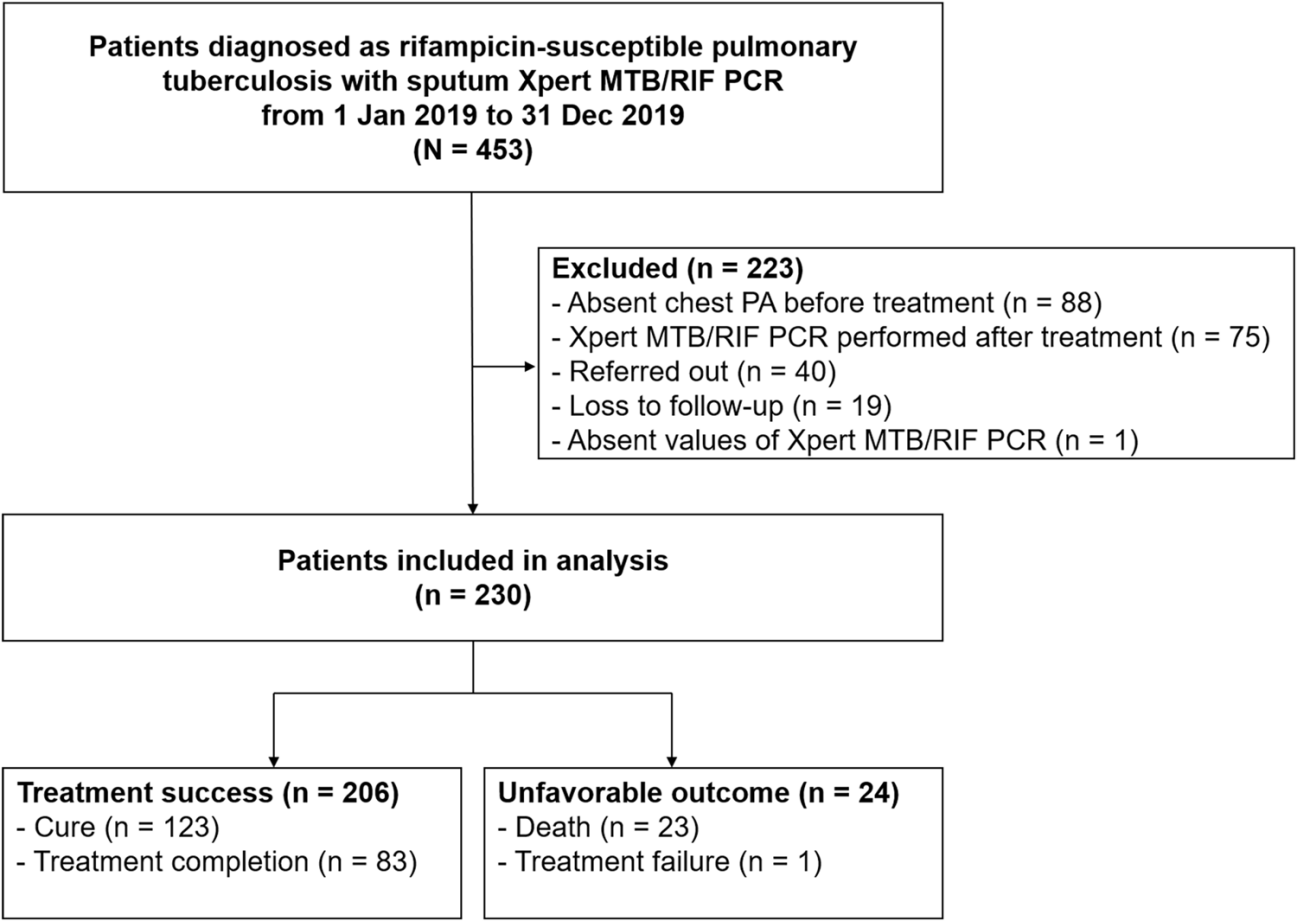
Flowchart of patient selection. PCR, polymerase chain reaction.

In accordance with the inclusion criteria, 453 patients underwent screening for rifampicin-susceptible pulmonary tuberculosis, identified by positive sputum samples according to the Xpert MTB/RIF assay. Following the application of the predefined exclusion criteria, 230 patients were selected for the final analysis. Of these, 206 (89.6%) achieved treatment success (Fig. 1).

### Baseline characteristics

The median age of the patients was 61.0 years, with a male predominance (76.1%). Sixty-three of 230 patients (27.4%) had a prior diagnosis and treatment for tuberculosis. The most prevalent comorbidities included hypertension, diabetes mellitus, and solid organ malignancy. Patients who achieved treatment success tended to be younger (median age: 59.0 vs. 75.0 years, *P* = 0.002) and had a higher BMI (median: 21.8 vs. 17.9 kg/m^2^, *P* = 0.002). No statistically significant differences were observed in other demographic factors, such as sex, smoking history, drinking history, and underlying comorbidities between the two groups (Table 1 and Supplementary Table S2).

**Table 1:** Baseline characteristics of the patients enrolled in the analysis.

Variable	Total N = 230	Treatment success n = 206	Unfavorable outcome n = 24	*P*
Age (years)	61.0 (51.0–76. 0)	59. 0 (50.0–75.0)	75.0 (63.5–81.0)	0.002
Male sex	175 (76.1)	153 (74.3)	22 (91. 7)	0.059
BMI (kg/m^2^)*	21.5 (18.8–23.8)	21.8 (19.3–23.8)	17.9 (16.4–22.0)	0.002
Ever smoker	130 (56.5)	115 (55.8)	15 (62.5)	0.532
Drinking history				0.656
Social drinker	105 (45.9)	94 (45.9)	11 (45.8)	
Binge drinker	26 (11.4)	22 (10.7)	4 (16.7)	
Unknown	98 (42.8)	89 (43.4)	9 (37.5)	
History of tuberculosis	63 (27.4)	54 (26.2)	9 (37.5)	0.241
Comorbidities				
Hypertension	74 (32.2)	65 (31.6)	9 (37.5)	0.555
Diabetes mellitus	64 (27.8)	58 (28.2)	6 (25.0)	0.744
Solid organ malignancy within 5 years	32 (13.9)	26 (12.6)	6 (25.0)	0.097
COPD	15 (6.5)	14 (6.8)	1 (4.2)	> 0.999
Asthma	11 (4.8)	11 (5.3)	0 (0.0)	0.611
Solid organ transplant	6 (2.6)	5 (2.4)	1 (4.2)	0.488
Immune suppressants	4 (1.7)	3 (1.5)	1 (4.2)	0.358
Hematologic malignancy within 5 years	4 (1.7)	2 (1.0)	2 (8.3)	0.055

*A total of 211 patients were included in the analysis of BMI, including 187 for treatment success assessment and 24 for unfavorable outcome assessment. *P*-values were calculated using the independent-samples t-test, Wilcoxon rank-sum test, the chi square test, or Fisher’s exact test, as appropriate. Numbers are presented as count (percentage) or median (interquartile range). BMI, body mass index; COPD, chronic obstructive pulmonary disease.

### Evaluation of sputum samples and radiographic findings

The overall smear-positive rate of the sputum samples was 49.6%. The culture positivity rates were 79.0% in liquid medium and 76.1% in solid medium. The median and minimum Ct values for the Xpert MTB/RIF assay were 26.2 and 25.0, respectively. These values did not show a statistically significant difference in relation to treatment success (Supplementary Table S2).

**Figure 2:**
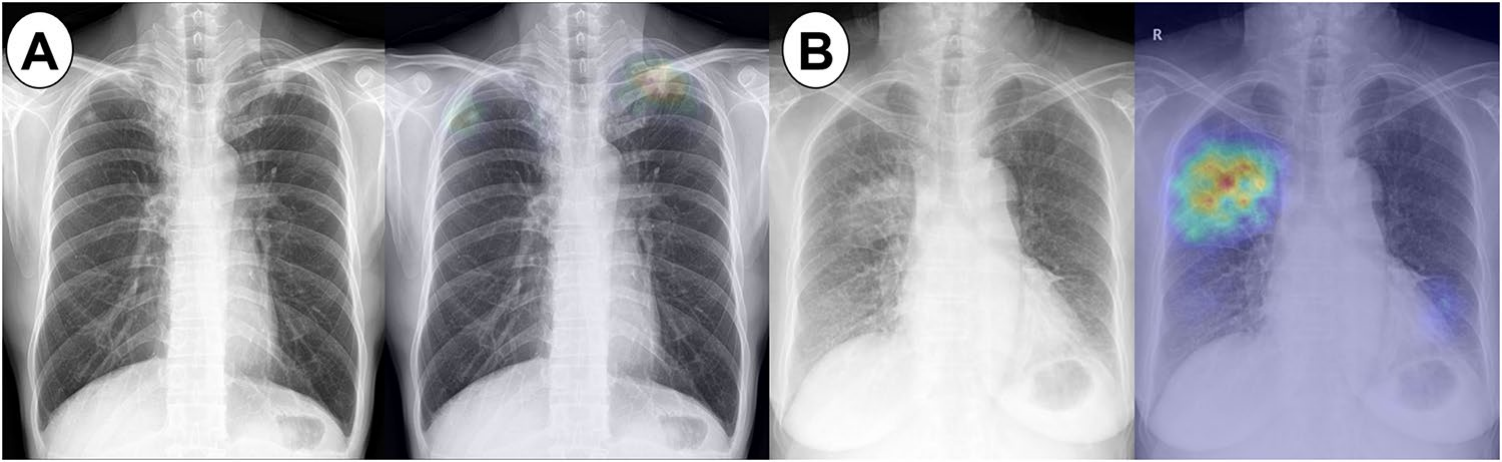
Representative chest X-ray images of patients with pulmonary tuberculosis. (**A**) Chest X-ray of a 48-year-old male patient with a tuberculosis extent score of 3.7. This patient was cured following a 6-month treatment course with the standard regimen*. (**B**) Chest X-ray of a 71-year-old female patient with a tuberculosis extent score of 16.8. The patient died during the course of the standard treatment regimen*. *Standard regimen refers to the combination of isoniazid, rifampicin, ethambutol, and pyrazinamide.

Cavitation was observed in 60 of 230 patients (26.1%), and the median tuberculosis extent score was 7.5 (IQR 2.9–14.4). The tuberculosis extent score was higher in patients with unfavorable outcomes (median 11.8, IQR 5.9–19.6) than in those with treatment success (median 7.2, IQR 2.8–13.3) (*P* = 0.021) (Supplementary Table S2). Representative chest X-rays of patients with treatment success and unfavorable outcomes are shown in Fig. 2.

### Factors associated with treatment success

An exploration of the association between clinical variables and treatment success was conducted. The univariate analysis revealed that younger age, higher BMI, absence of hematologic malignancy within the past 5 years, and a lower tuberculosis extent score were associated with treatment success. Following stepwise selection and multivariate analysis, the factors significantly associated with treatment success included younger age (OR 0.942, 95% CI 0.910–0.974), female sex (OR 6.799, 95% CI 1.214–38.092), higher BMI (OR 1.204, 95% CI 1.033–1.403), absence of hematologic malignancy (OR 0.069, 95% CI 0.007–0.723), and a lower tuberculosis extent score (OR 0.938, 95% CI 0.895–0.983). However, the median (OR 1.032, 95% CI 0.955–1.115) and minimum (OR 1.033, 95% CI 0.959–1.113) Ct values were not associated with treatment success (Table 2).

**Table 2:** Factors associated with treatment success.

Variables	Unadjusted OR (95% CI)	Adjusted OR (95% CI)
Age	0.957 (0.928–0.986)*	0.942 (0.910–0.974)*
Female sex	3.810 (0.867–16.753)	6.799 (1.214–38.092)*
Body mass index	1.231 (1.067–1.420)*	1.204 (1.033–1.403)*
Ever smoker	0.773 (0.324–1.848)	
Drinking history		
Social drinker	Reference	
Binge drinker	0.745 (0.278–1.996)	
History of TB	0.592 (0.245–1.432)	
Asthma	2.885 (0.145–57.256)	
COPD	1.181 (0.197–7.065)	
Bronchiectasis	0.348 (0.044–2.754)	
AIDS	0.842 (0.027–26.426)	
Diabetes mellitus	1.176 (0.445–3.109)	
Hypertension	0.768 (0.320–1.847)	
Immune suppressants	0.270 (0.030–2.387)	
Solid organ transplant	0.428 (0.058–3.152)	
Hematologic malignancy within 5 years	0.110 (0.015–0.819)*	0.069 (0.007–0.723)*
Solid organ malignancy within 5 years	0.433 (0.158–1.191)	–
Median Ct value	1.032 (0.955–1.115)	–
Minimum Ct value	1.033 (0.959–1.113)	–
TB extent (%)	0.951 (0.913–0.990) *	0.938 (0.895–0.983)*

*P < 0.05. OR, odds ratio; CI, confidence interval; TB, tuberculosis; COPD, chronic obstructive pulmonary disease; AIDS, acquired immune deficiency syndrome; Ct, cycle threshold.

### Factors associated with the negative conversion of sputum culture at 8 weeks after treatment

**Figure 3:**
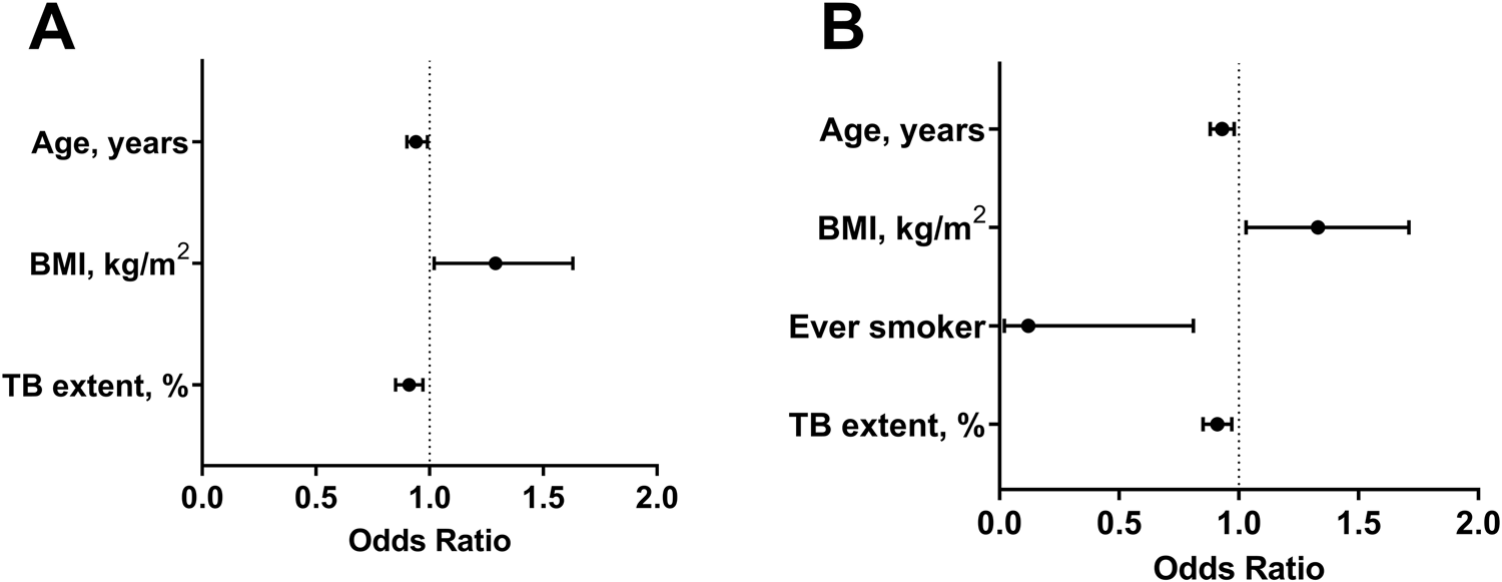
Factors associated with negative culture conversion at 8 weeks after treatment. The forest plots reveal the variables that were significantly associated with negative culture conversion at 8 weeks after treatment in liquid (**A**) and solid (**B**) media. BMI, body mass index; TB, tuberculosis.

The factors associated with culture conversion at 8 weeks after treatment in both liquid and solid media were evaluated. Among the 177 patients with baseline positive culture results in liquid medium, 175 had known microbiological outcomes at 8 weeks after treatment. After multivariate adjustment, younger age (OR 0.940, 95% CI 0.895–0.986), higher BMI (OR 1.289, 95% CI 1.023–1.625), and lower tuberculosis extent score (OR 0.911, 95% CI 0.853–0.973) were significantly associated with a higher rate of negative culture conversion at 8 weeks after treatment (Fig. 3A). The median (OR 1.029, 95% CI 0.920–1.152) and minimum (OR 1.029, 95% CI 0.923–1.147) Ct values were not associated with outcomes (Supplementary Table S3).

Of the 169 patients with positive baseline culture results in solid medium, 167 were evaluated for known microbiological outcomes at 8 weeks after treatment. The factors associated with negative culture conversion in solid medium were similar to those in liquid medium, and included younger age (OR 0.930, 95% CI 0.884–0.979), higher BMI (OR 1.326, 95% CI 1.028–1.710), and lower tuberculosis extent score (OR 0.910, 95% CI 0.850–0.973). Additionally, the presence of any smoking history was associated with a lower rate of culture conversion in solid medium (OR 0.116, 95% CI 0.017–0.814) (Fig. 3B). The median (OR 1.051, 95% CI 0.942–1.173) and minimum (OR 1.050, 95% CI 0.944–1.168) Ct values were not associated with outcomes (Supplementary Table S4).

## Discussion

This study, which was conducted across six referral centers in South Korea, provides significant insights into the factors associated with treatment success and culture conversion in the treatment of pulmonary tuberculosis. The findings highlight the emerging role of chest imaging in tuberculosis diagnosis and management, particularly through the use of AI-based models to calculate tuberculosis extent scores from chest X-ray. These AI-based scores are numerical values that can be used to objectively evaluate the radiographic severity of pulmonary tuberculosis at diagnosis, underscoring the potential of AI in predicting treatment outcomes. Notably, the present study found that the representative Ct values from the Xpert MTB/RIF assay at diagnosis were not significantly correlated with either treatment success or culture conversion rate at 8 weeks after treatment.

The use of AI in the diagnosis of pulmonary tuberculosis has been a subject of extensive research. A systematic review by Harris et al.^23^ analyzed 53 studies and found that AI demonstrated a high diagnostic accuracy. The review reported an AUC of 0.88 for development studies and 0.75 for clinical studies, indicating the strong ability of AI to distinguish between patients with and without tuberculosis. Further advances in AI have led to the development of deep learning-based automatic detection models that have shown exceptional classification performance, with AUCs ranging from 0.977 to 1.000. These models have demonstrated superior accuracy over physicians for identifying patients with tuberculosis^24^. Additionally, a recent deep learning-based software has been validated for its accuracy, achieving a sensitivity of 0.93, which meets the criteria for World Health Organization-recommended triage tests^25^. Notably, however, these studies have primarily focused on the diagnostic aspect of pulmonary tuberculosis, relying on microbiological confirmation as the gold standard for final diagnosis. The application value of AI for tuberculosis diagnosis is evident, but its role extends beyond the mere identification of tuberculosis; it may also be useful for predicting the treatment effect, which is crucial for comprehensive tuberculosis care.

One key finding of the present study is the importance of radiographic findings in managing pulmonary tuberculosis. Radiographic findings, such as bilateral involvement, presence of cavities, and multi-lobar involvement, are well-known risk factors for a poor prognosis in patients with tuberculosis^26,27^. However, these factors are difficult to quantify and integrate into daily practice. The tuberculosis extent score, which can be quantified using a previously developed AI-based prognostic model^8^, can be used as a numerical indicator of tuberculosis severity. This finding aligns with previous studies on patients with non-tuberculous mycobacterial pulmonary disease and highlights the potential of radiographic assessments for guiding tuberculosis treatment^15,28^. Advances in AI applied to radiological data present new opportunities for enhancing tuberculosis prognostication. For instance, patients with a higher tuberculosis extent score, which indicates more severe disease, may require more rigorous monitoring and adherence to their prescribed medication regimen, while patients with a lower tuberculosis extent score may be suitable for shorter regimens^29^. The precise assessment of disease severity can help to ensure that patients are receiving the appropriate level of care and attention. Moreover, these patients might also benefit from extended treatment durations. Typically, the tuberculosis treatment duration is standardized^1^; however, patients with more severe disease at diagnosis, as indicated by a higher tuberculosis extent score, might require a longer treatment duration to fully eradicate the infection.

Interestingly, the Ct values obtained from the Xpert MTB/RIF assay, which represent the bacillary burden in the sputum^11^, did not show a statistically significant difference in relation to the treatment success or culture conversion rate at 8 weeks after treatment. This finding suggests that while Ct values are useful for diagnosing tuberculosis and detecting rifampin resistance and earlier bacterial clearance, they may not be reliable predictors of final treatment outcomes. This is similar to a previous study by Namugenyi et al.^13^, who showed that Ct values are associated with the achievement of culture conversion at weeks 2 and 4 after treatment, but not at week 8. The bacterial burden of tuberculosis rapidly decreases during the early course of treatment^30^, which may explain why the Ct value itself did not have a critical influence on the final treatment outcomes. This observation is crucial for clinicians because it indicates that while Ct values are valuable diagnostic tools, they should not be solely relied upon for predicting treatment success.

In addition to these novel findings, the present study reinforces the significance of individual patient factors in determining tuberculosis treatment outcomes. Younger patients and those with a higher BMI, which is indicative of a better nutritional status, tend to have more favorable responses to tuberculosis treatment^31^. This finding is in line with other previous research emphasizing the critical role of nutritional status in tuberculosis recovery and the need for comprehensive patient care addressing nutritional needs^32^. The absence of hematologic malignancy in the previous 5 years has also been linked to better treatment outcomes, highlighting the intricate relationship between tuberculosis and hematologic malignancy^33^. Another study showed that a history of smoking is associated with delayed culture conversion in solid medium, highlighting the detrimental impact of smoking on tuberculosis treatment outcomes^34^.

The strengths of this study lie in its multicenter approach: it was conducted across six diverse regions of South Korea, which enhances the generalizability of the findings. The utilization of AI-based models, which offer a quantifiable measure of the radiographic severity of the disease at diagnosis, are a significant step forward from traditional methods. However, this study is not without limitations. Given that the study was retrospective in nature and it was conducted at referral centers, the findings might have limited applicability to the primary care setting or other regions. Future research could address these limitations by incorporating prospective designs and expanding the inclusion to primary care settings. Moreover, this study evaluated the Ct values of Xpert MTB/RIF assays exclusively. The relevance of other RT-PCR Ct values merits further exploration. Additionally, the prediction performance may vary with the AI models used. Comparing various AI-based models could prove beneficial.

## Conclusions

This study demonstrates that the AI-based radiographic severity score assessed at the time of pulmonary tuberculosis diagnosis is significantly associated with treatment success and culture conversion at 8 weeks after treatment. These findings underscore the potential of AI in enhancing the prognostication and management of pulmonary tuberculosis. Future research should focus on exploring how these prognostic insights can be integrated into personalized patient management strategies.

### Supplementary Information

Supplementary Tables.

## Data Availability

The data utilized in this analysis are available from the corresponding author upon reasonable request.
