# Supplementary material for: Artificial intelligence-based radiographic extent analysis to predict tuberculosis treatment outcomes: a multicenter cohort study
Source: Sci Rep. 2024 Jun 7;14:13162. doi: 10.1038/s41598-024-63885-0 (PMC11161500; doi:10.1038/s41598-024-63885-0)
Supplement: Supplementary file 1 — Supplementary Tables. [file 41598_2024_63885_MOESM1_ESM.docx]

**Supplementary Material**

**Supplementary Table S1. Protocol Numbers of the Institutional Review Board of Each Center**

| Center | Protocol number |
| --- | --- |
| Seoul National University Bundang Hospital | B-2208-773-404 |
| Seoul National University Hospital | H-2206-179-1336 |
| National Medical Center | NMC-2022-08-092 |
| Severance Hospital | 4-2022-0820 |
| Pusan National University Hospital | 2207-025-117 |
| Pusan National University Yangsan Hospital | 05-2022-184 |

**Supplementary Table S2. Additional Characteristics of the Patients Enrolled in the Analysis**

| Variable | Total  N=230 | Treatment success  n=206 | Unfavorable outcome  n=24 | *P* |
| --- | --- | --- | --- | --- |
| Comorbidities | 11 (4.8) | 11 (5.3) | 0 (0.0) | 0.611 |
| Bronchiectasis | 5 (2.2) | 4 (1.9) | 1 (4.2) | 0.427 |
| AIDS | 3 (1.3) | 3 (1.5) | 0 (0.0) | >0.999 |
| Sputum smear positive | 110 (49.6) | 97 (48.7) | 13 (56.5) | 0.480 |
| Liquid media culture positive | 177 (79.0) | 161 (80.5) | 16 (66.7) | 0.116 |
| Solid media culture positive | 169 (76.1) | 151 (76.3) | 18 (75.0) | 0.891 |
| Xpert Ct value, median | 26.2 (21.3–29.0) | 26.2 (21.3–29.1) | 25.5 (18.7–28.3) | 0.442 |
| Xpert Ct value, minimum | 25.0 (20.0–28.1) | 25.1 (20.2–28.1) | 24.5 (17.5–27.2) | 0.396 |
| Presence of cavities | 60 (26.1) | 50 (24.3) | 10 (41.7) | 0.066 |
| Tuberculosis extent (%) | 7.5 (2.9–14.4) | 7.2 (2.8–13.3) | 11.8 (5.9–19.6) | 0.021 |

^*^A total of 211 patients were included in the analysis of BMI, including 187 for treatment success assessment and 24 for unfavorable outcome assessment. *P*-values were calculated using the independent-samples t-test, Wilcoxon rank-sum test, the chi square test, or Fisher’s exact test, as appropriate. Numbers are presented as count (percentage) or median (interquartile range). Abbreviation: AIDS, acquired immune deficiency syndrome; Ct, cycle threshold.

**Supplementary Table S3. Factors Associated with Negative Culture Conversion at 8 Weeks After Treatment in Liquid Medium**

| Variables | Unadjusted OR  (95% CI) | Adjusted OR  (95% CI) |
| --- | --- | --- |
| Age | 0.952 (0.911–0.995) ^*^ | 0.940 (0.895–0.986) ^*^ |
| Female sex | 3.667 (0.456–29.480) | - |
| Body mass index | 1.296 (1.045–1.609) ^*^ | 1.289 (1.023–1.625) ^*^ |
| Ever smoker | 0.456 (0.117–1.781) | - |
| Drinking history |  |  |
| Social drinker | Reference |  |
| Binge drinker | 1.049 (0.216–5.099) | - |
| History of TB | 0.697 (0.176–2.771) | - |
| Asthma | 1.248 (0.057–27.249) | - |
| COPD | 1.248 (0.057–27.249) | - |
| Bronchiectasis | 0.354 (0.008–15.319) | - |
| AIDS | 0.354 (0.008–15.319) | - |
| Diabetes mellitus | 1.136 (0.289–4.464) | - |
| Hypertension | 0.790 (0.221–2.818) | - |
| Immune suppressants | 0.354 (0.008–15.319) | - |
| Solid organ transplant | 0.645 (0.023–17.838) | - |
| Hematologic malignancy within 5 years | 0.354 (0.008–15.319) | - |
| Solid organ malignancy within 5 years | 0.391 (0.096–1.593) | - |
| Median Ct value | 1.029 (0.920–1.152) | - |
| Minimum Ct value | 1.029 (0.923–1.147) | - |
| TB extent (%) | 0.912 (0.864–0.964) ^*^ | 0.911 (0.853–0.973) ^*^ |

**P* < 0.05. Abbreviations: OR, odds ratio; CI, confidence interval; TB, tuberculosis; COPD, chronic obstructive pulmonary disease; AIDS, acquired immune deficiency syndrome; Ct, cycle threshold.

**Supplementary Table S4. Factors Associated with Negative Culture Conversion at 8 Weeks After Treatment in Solid Medium**

| Variables | Unadjusted OR  (95% CI) | Adjusted OR  (95% CI) |
| --- | --- | --- |
| Age | 0.956 (0.915–0.999) ^*^ | 0.930 (0.884–0.979) ^*^ |
| Female sex | 9.386 (0.525–167.689) | - |
| Body mass index | 1.357 (1.082–1.702) ^*^ | 1.326 (1.028–1.710) ^*^ |
| Ever smoker | 0.246 (0.052–1.177) | 0.116 (0.017–0.814) ^*^ |
| Drinking history |  |  |
| Social drinker | Reference |  |
| Binge drinker | 1.027 (0.211–5.009) | - |
| History of TB | 0.715 (0.180–2.846) | - |
| Asthma | 1.481 (0.070–31.498) | - |
| COPD | 0.994 (0.042–23.532) | - |
| Bronchiectasis | 0.372 (0.009–16.118) | - |
| AIDS | 0.225 (0.002–21.945) | - |
| Diabetes mellitus | 2.186 (0.456–10.487) | - |
| Hypertension | 1.295 (0.330–5.089) | - |
| Immune suppressants | 0.372 (0.009–16.118) | - |
| Solid organ transplant | 0.678 (0.025–18.780) | - |
| Hematologic malignancy within 5 years | 0.372 (0.009–16.118) | - |
| Solid organ malignancy within 5 years | 0.415 (0.102–1.689) | - |
| Median Ct value | 1.051 (0.942–1.173) | - |
| Minimum Ct value | 1.050 (0.944–1.168) | - |
| TB extent (%) | 0.923 (0.873–0.976) ^*^ | 0.910 (0.850–0.973) ^*^ |

**P* < 0.05. Abbreviations: OR, odds ratio; CI, confidence interval; TB, tuberculosis; COPD, chronic obstructive pulmonary disease; AIDS, acquired immune deficiency syndrome; Ct, cycle threshold.
